# Endoscope-Assisted Traversal of a Ureteral Stricture From Percutaneous Nephrostomy Access

**DOI:** 10.7759/cureus.30913

**Published:** 2022-10-31

**Authors:** Aaroh M Parikh, Darryl Smith, Rayhan Hai, Ajit Vyas

**Affiliations:** 1 Department of Radiology, Baylor College of Medicine, Houston, USA; 2 Interventional Radiology, Michael E. DeBakey Veterans Affairs Medical Center, Houston, USA

**Keywords:** image guidance, percutaneous endoscopy, genitourinary endoscopy, endoscopy, interventional radiology

## Abstract

Interventional radiology-operated endoscopy has a small but growing number of applications. In this clinical case report, we describe the use of an endoscope to assist the traversal of a high-grade ureteral stricture from percutaneous nephrostomy (i.e., antegrade) access. Direct visualization of the stricture allowed the identification of a central channel that was not present in fluoroscopic images, making endoscopy essential to the technical success of the procedure. Endoscopy is a powerful adjunct to image-guided techniques, particularly in challenging interventions or cases with complex anatomy.

## Introduction

Endoscopy provides valuable information to guide both diagnostic and therapeutic procedures. Direct visualization can be a powerful adjunct to image-guided techniques, particularly in complex or challenging interventions. The first documented use of endoscopy in the interventional radiology suite was reported by Yamakawa et al. [1], although its usage was limited to the retrieval of biliary calculi. Recent advances in imaging and endoscopy technology (in particular the development of appropriately sized endoscopes for use through percutaneous access) have spurred a resurgence in interest. Although the role of endoscopy has been very limited in interventional radiology, a small but growing number of applications have emerged. This report presents a case of interventional radiology-operated endoscopy used to traverse a ureteral stricture from percutaneous nephrostomy (PCN; i.e., antegrade) access.

## Case presentation

Our patient was a 65-year-old male with a history of stage IV bladder cancer and stage II prostate cancer, status post-robotic radical cystoprostatectomy, and creation of a right lower quadrant ileal conduit at an outside tertiary care cancer center. Following his initial surgery, a left ureteral stent was placed and subsequently removed after approximately six months. He later sought care at our institution, where he was found to have a mild right and severe left hydronephrosis. Interventional radiology was consulted for the decompression of the left collecting system.

**Figure 1:**
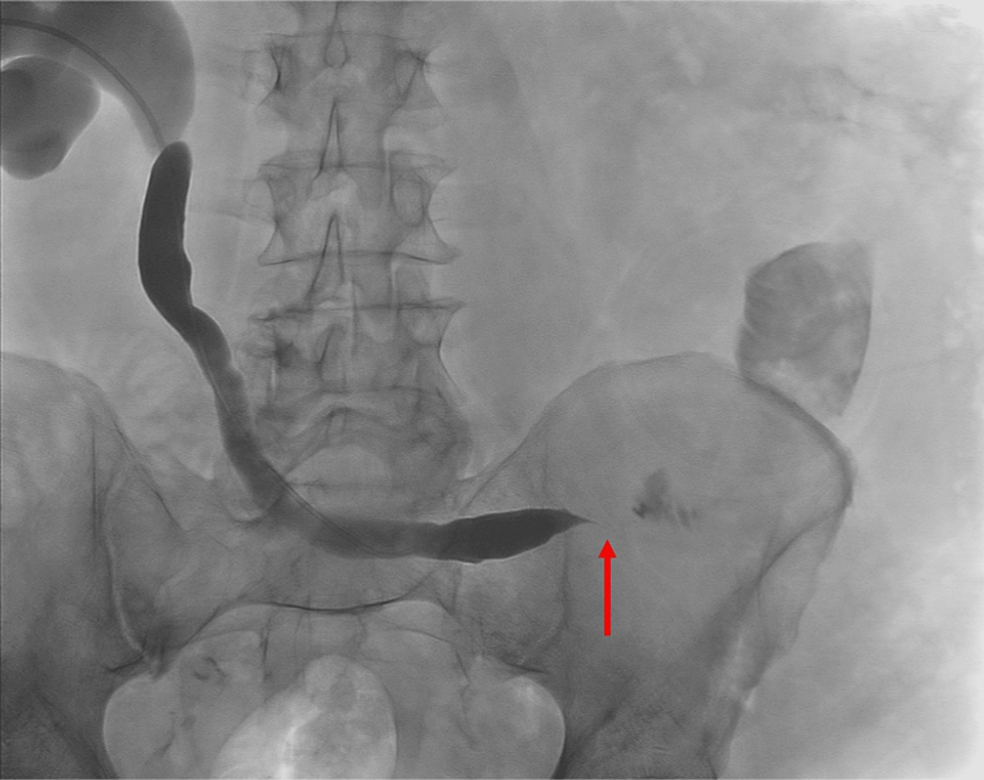
Distal ureteral stricture. An antegrade nephrostogram obtained in a prone position during the index procedure showed left hydroureteronephrosis and a high-grade stricture in the distal ureter. There was opacification of the ileal conduit via a very narrow channel (arrow), indicating patency of the stricture and surgical anastomosis.

During the index procedure, the left kidney was accessed percutaneously in the usual fashion under image guidance. Antegrade nephrostogram confirmed severe hydronephrosis and revealed a focal, high-grade stricture in the distal ureter; however, there was a passage of contrast distally into the ileal conduit, indicating patency of the distal ureter and surgical anastomosis (Figure 1). Despite multiple attempts, the stricture could not be traversed to place a percutaneous nephroureterostomy (PCNU) tube. As a result, a PCN tube was placed instead. Approximately eight weeks after the initial placement, the PCN was upsized from 8.5 to 12 French.

**Figure 2:**
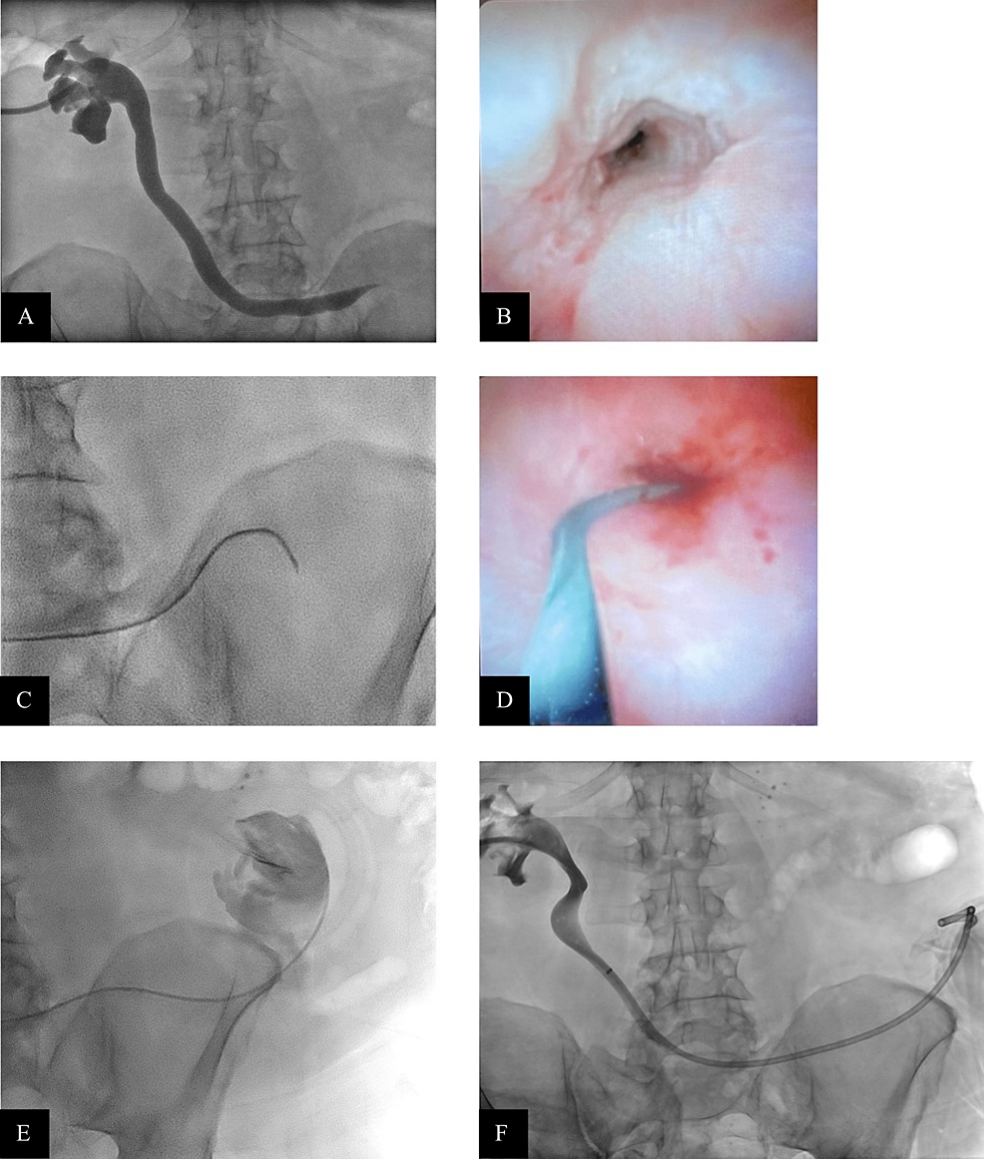
Endoscopy-assisted traversal of the ureteral stricture. An antegrade nephrostogram obtained in a prone position during the endoscopy-assisted procedure (A) redemonstrated the stricture in the distal ureter but did not show the passage of contrast distally into the ileal conduit. Endoscopy of the stricture (B) revealed a central channel. Multiple wire and catheter combinations were attempted to traverse the stricture (C and D). After successful traversal, contrast injection (E) confirmed access into the ileal conduit. Following balloon dilation of the stricture, a 10-French × 26 cm PCNU tube was placed (F). PCNU, percutaneous nephroureterostomy

Approximately 3.5 months after the initial placement, the patient was brought back to attempt the conversion of the PCN to a PCNU with aid of an endoscope to directly visualize the ureteral stricture. Preprocedure serum creatinine was 1.49 mg/dL and blood urea nitrogen was 22 mg/dL. The complete blood count was within normal limits. Because this procedure would require the patient to lie prone for a prolonged period, the procedure was performed under general anesthesia. An initial antegrade nephrostogram obtained through the indwelling 12-French PCN tube redemonstrated the stricture in the distal ureter. The indwelling PCN was exchanged for a 12-French sheath over a wire. The endoscope (SpyGlass DS System, Boston Scientific, Marlborough, MA, USA) was introduced through the sheath and advanced to the distal ureter. On fluoroscopic images, contrast did not pass distal to the stricture into the ileal conduit (Figure 2A). However, in real-time endoscopic images, a central channel was visualized at the site of the stricture (Figure 2B). In addition to providing direct visualization, the endoscope also allowed the instillation of saline to distend the distal ureter and atraumatically probe the stenotic channel.

Numerous attempts were made to traverse the stricture using various wire and catheter combinations (Figure 2C-2D). Ultimately, success was achieved with the combination of a 5-French hydrophilic catheter (Glidecath, Terumo Interventional Systems, Somerset, NJ, USA) and a 0.035-inch stiff hydrophilic wire (Stiff Glidewire, Terumo Interventional Systems). Contrast injection confirmed access into the ileal conduit (Figure 2E). Balloon dilation of the stricture was then performed using a 3 mm × 40 mm balloon (EverCross, Medtronic, Minneapolis, MN, USA) followed by a 6 mm × 40 mm balloon (Dorado, BD, Franklin Lakes, NJ, USA) over a 0.035-inch Amplatz wire (Boston Scientific). The balloon was then exchanged over the wire for a 10-French × 26 cm PCNU tube, with the distal tip positioned within the ileal conduit (Figure 2F). The proximal pigtail of the PCNU tube was deliberately left unformed to minimize the risk of retracting the distal loop through the ureteral stricture. Contrast injection confirmed excellent positioning. The patient tolerated the procedure well. There were no procedural complications.

There was low suspicion of recurrent malignancy at the site of the stricture, as a PET/CT performed eight weeks before the procedure showed no evidence of recurrent, residual, or metastatic disease. As a result, a biopsy of the stricture was not performed. After discharge, the patient was referred back to the urology service for further management and possible internalization of the PCNU or conversion to a urinary diversion catheter. If no intervention was planned, we would exchange the PCNU catheter every six to eight weeks, at which point follow-up laboratory studies would be obtained. Nevertheless, no change in serum chemistries or renal function was expected, as the patient’s urine remained diverted following the procedure.

## Discussion

The use of endoscopy in interventional radiology has traditionally been limited to a small number of indications and performed at only a handful of institutions. The primary benefit of endoscopy as an adjunct to image-guided techniques is the addition of direct visualization, which is especially useful in challenging interventions. Technical success in such procedures provides significant value to patients and obviates more invasive or complex interventions. Furthermore, the use of endoscopy may reduce fluoroscopy time and radiation dose to both the patient and the operator [2].

The typical indications for interventional radiology-operated endoscopy concern the management of biliary diseases, such as gallstone retrieval or choledochoscopy [3]. However, the potential applications of endoscopy in interventional radiology extend beyond the biliary system. In the gastrointestinal tract, combined image-guided and endoscopic techniques have been used by interventional radiologists to (re)place percutaneous gastrojejunostomy tubes, place endoluminal stents across obstructing lesions, and retrieve foreign bodies through percutaneous access [4-7]. Interventional radiology-operated endoscopy has also been reported in musculoskeletal procedures, specifically in treating synovial chondromatosis and sequestered intervertebral disc herniation [8,9].

In the genitourinary system, interventional radiology is routinely consulted for PCN placement in patients with obstructing calculi to enable subsequent nephrolithotripsy. However, the definitive endoscopy-assisted procedure is usually performed by urology. Only a few applications of interventional radiology-operated endoscopy have been reported in the genitourinary system. Chick et al reported the use of a disposable ureteroscope to place a stent across the stenotic anastomosis of a pyelovesicostomy in a renal transplant patient [10]. Healey et al. reported a case similar to ours, in which endoscopy was used via ileal conduit (i.e., retrograde) access to cannulate the ureters and place bilateral ureteronephric stents [4]. Nephroscopy has also been used to assist in the retrieval of migrated renal artery embolization coils [11,12].

In this report, we present a case of interventional radiology-operated endoscopy to aid the traversal of a high-grade ureteral stricture and the conversion of a PCN to a PCNU. The use of endoscopy was critical to the technical success of the procedure. Intraluminal imaging provided an orthogonal cross-sectional plane to the typical coronal projection seen during fluoroscopy. Contrast injection under fluoroscopy failed to demonstrate patency of the distal ureter. Without the benefit of real-time direct visualization, a central tract would not have been identified and attempts to traverse the stricture would have likely failed. Thus, this case is particularly illustrative of the benefits of interventional radiology-operated endoscopy. In challenging cases that require the traversal of a stricture, balloon dilation, or placement of a nephroureteral stent, the combination of image guidance and direct visualization can be especially useful. Although interventional radiology has collaborated with urology in joint cases utilizing endoscopy, the primary operator (i.e., endoscopist) has typically been the urologist. In this case report, the interventional radiologist as the primary operator of the endoscope represents a shift in that paradigm. Interventional radiology-operated endoscopy likely reduces the total procedure time and time to scheduling, as coordination with another service is not necessary.

Certain technical considerations must be made when evaluating a patient for endoscopy-assisted intervention. The location of percutaneous access should be selected based on the relevant anatomy while bearing in mind the ergonomics of using an endoscope. Choosing the length and diameter of the endoscope requires an understanding of its relative advantages and disadvantages. Although a longer endoscope enables more distal access, it can also be more challenging to navigate and manipulate in locations very distal to the access site. Whereas endoscopes with large diameters offer a larger working channel, a general principle is to select the smallest channel able to achieve technical success. Endoscopes also vary in their rigidity; for example, a flexible endoscope is preferred in distal genitourinary cases such as ours. Because endoscopy requires a well-formed tract, it is customary to wait four to six weeks after initial access before performing the endoscopy-assisted intervention in cases of infection, renal compromise, or massive dilatation of the collecting system. General anesthesia is preferred, especially for prolonged interventions or those that require prone positioning. Lastly, appropriate patient selection with multispecialty collaboration is paramount.

Familiarity with the equipment required for endoscopy may promote better acceptance of endoscopic techniques among interventional radiologists. The endoscope is part of a portable endoscopy tower. The tower can be connected to the existing display in the angiography suite, allowing both fluoroscopic and endoscopic images to be displayed on the same monitor. Endoscopic images can also be saved and archived alongside fluoroscopic images from the procedure. Endoscopy requires saline irrigation and aspiration, which are performed through dedicated channels in the endoscope and can be controlled by the operator. Many modern endoscopes, such as SpyGlass used in our case, allow four-way articulation to direct and maneuver the endoscope in different directions. Interventional radiology-operated endoscopy has a small learning curve due to the readily translatable skills practiced in similar image-guided techniques.

## Conclusions

In the hands of an interventional radiologist, endoscopy is a valuable adjunct to image-guided techniques. We present a case in which endoscopy was used to assist the traversal of a high-grade ureteral stricture from PCN access. The direct visualization provided by the endoscope was essential to the technical success of the procedure. Interventional radiology-operated endoscopy is a useful asset in challenging interventions and provides meaningful value to patients and operators alike.
